# Gymkhana at Cardiff

**Published:** 1919-07-19

**Authors:** 


### Gymkhana at Cardiff.

Through the kindness of Mr. C. C. Williams, a gymkhana was held in the Park of Llanrumney Hall on the 9th inst., to benefit the King Edward VII. Hospital at Cardiff, and, after the weariness of war restrictions, it possessed all the attractions of novelty. The event proved a great success in every way. The entries included well-known county people, and the prize-givers Lord Tredegar and Lord Glanely, the winner of this year's Derby. In addition to the horse and foot races, there were roundabouts, gymnasts, a very popular palmist and several side-shows, and music was supplied by a quite delightful band of little boys from the Industrial School at Treharris. A profitable feature was the tea tent, where some £40 was realised by the energy and businesslike ability of a devoted band of Cardiff ladies. Altogether, the gymkhana is estimated to realise at least £150. Despite the difficulties of access to Llanrumney from Cardiff, there was probably an attendance of nearly 2,000 people. Easier access would have assuredly trebled the number.

